# Prognosis of Paralysis Occurring in Children Determined by Electric Examination

**Published:** 1887-09

**Authors:** E. C. Smith

**Affiliations:** Richmond, Va.


### PROGNOSIS OF PARALYSIS OCCURRING IN CHILDREN DETERMINED BY ELECTRIC EXAMINATION.

BY E. C. SMITH, B. SC., M. D., RICHMOND, VA.

I do not intend to limit myself to the consideration of the prognosis of Poliomyelitis anterior (true infanti e paralysis of MM. Charcot and Joffroy), but to the prognosis of paralysis occurring in children, be it of cerebral, spinal, or peripheral origin, determined by electric contractility, as illustrated by the following cases:

Case I. This is a boy, æt. 7, with partial motor paralysis of his right leg, dating from birth, who was brought to me for treatment for acquired talipes equino varus. Upon electric examination I found but feeble contractility of the muscles of the leg. There was shortening of about an inch in the affected limb, and trophic lesions were well marked. The faradic induced current was applied to the weakened muscles three times a week for a period of three months, the labile application, with the sponge alternating with the stabile application, with the metallic electrodes, from ten to twenty minutes. The result was excellent. The foot was brought back into its proper position so soon as the weakened muscles had regained their strength; and, although it has been four years since the patient was discharged, there has been no reappearance of the trouble. The little fellow grows stronger daily from his ability to use each muscle properly when he walks. He wears a shoe with a cork sole sufficient to make up for his congenital shortening, if I may so call it, which shortening has not progressed since the suspension of treatment. The atrophied limb now keeps pace in growth with his other.

Case II. A boy five years old, with hemiphlegia of the right side. He had never walked. Gave a history of convulsions at an early age. There was marked contractions of the right arm, and the corresponding leg was slightly drawn. Trophic lesions were well marked in both upper and lower extremity. Electric examination gave but slight response to the primary current and that only when quite powerful. Sensation was not impaired. Considerable nervous manifestations were present. In treating this case strychnine was given, *per orem*, in gradually increasing doses, three times a day, at intervals of three or four weeks. For the first five months I saw him three times a week and applied central galvanization descending, and peripheral faradization, primary. At the end of this time he was brought to my office once a week for a period of four months longer, when he was discharged, able to walk anywhere and with a grip in his right hand sufficient to carry an ordinary bag of books.

Case III. A boy four years old now under my care with motor paraphelegia. No trophic lesions, have, as yet, developed. Sensibility is unimpaired. Electric examination gave no response with either the induced or primary faradic current. The prognosis was considered very doubtful, yet, as a *dernier resort*, he was, for three weeks, subjected to galvanization of his spine and extremities. I gave him in this time also, two hypodermic injections of strychnine sulphate, 1-120 and 1-60 of a grain. At the end of this period he began to respond to a strong primary faradic current. Since then weaker and weaker currents effect the same end, and he continues to improve.

Case IV. Lastly, a paraplegic patient, a boy five years old, was brought to me for treatment. Trophic symptoms were present in an aggravated degree and there were contractions in both limbs. I could elicit no reaction to the strongest induced or primary faradic. He was then, for two months, placed under galvanism, with strychnine internally. At the expiration of two months of such treatment the faradic current was still inert. Further treatment was abandoned in this case as one to which I was confident no benefit would accrue.

The cases here reported, as well as many others which might be adduced, seem to warrant the following conclusions as to the prognosis furnished by electric contractibility in paralysis of early life:

1st. A favorable prognosis may be given in all cases which respond to the *induced* faradic current without preliminary treatment.

2d. Those cases which respond to the *primary* faradic current without preliminary treatment can be benefited and perhaps very much.

3d. Those which respond to the *primary* faradic current after preliminary galvanization and strychnine may be improved to a greater or less extent.

4th. Those giving no response to the faradic after such preliminary use of the galvanic current, aided by strychnine internally, have been unimproved.—*Practice.*

